# Oncolytic Immunotherapy: Where Are We Clinically?

**DOI:** 10.1155/2014/862925

**Published:** 2014-01-16

**Authors:** Akseli Hemminki

**Affiliations:** ^1^Cancer Gene Therapy Group, Haartman Institute, University of Helsinki, Haartmaninkatu 3, 00290 Helsinki, Finland; ^2^TILT Biotherapeutics Ltd., P. Hesperiankatu 37A22, 00260 Helsinki, Finland

## Abstract

Following a century of preclinical and clinical work, oncolytic viruses are now proving themselves in randomized phase 3 trials. Interestingly, human data indicates that these agents have potent immunostimulatory activity, raising the possibility that the key consequence of oncolysis might be induction of antitumor immunity, especially in the context of viruses harboring immunostimulatory transgenes. While safety and efficacy of many types of oncolytic viruses, including adenovirus, herpes, reo, and vaccinia seem promising, few mechanisms of action studies have been performed with human substrates. Thus, the relative contribution of “pure” oncolysis, the immune response resulting from oncolysis, and the added benefit of adding a transgene remain poorly understood. Here, the available clinical data on oncolytic viruses is reviewed, with emphasis on immunological aspects.

Since the “War on Cancer” was launched in the 1970s, the treatment of most cancers has improved steadily. Nevertheless, most metastatic solid tumors remain incurable. Therefore, new agents with novel mechanisms of action and lacking cross-resistance to the currently available approaches are needed.

Due to hypothetical safety concerns, cancer gene therapy approaches have traditionally been based on viruses that are unable to replicate. Although such “vectors” have provided high preclinical efficacy and good clinical safety data, trials have suggested that their efficacy may be limited when faced with advanced and bulky disease, because of limited penetration from the needle tract into further area of the tumor. Nevertheless, in the context of local disease, even replication deficient viruses could have their uses when combined optimally to routine therapies [1, 2].

In the context of cancer therapy, nonreplicating viruses have largely been abandoned in favor of replication competent platforms, since there are few advantages to the former, as safety of the latter has proven excellent. Moreover, one could argue that there are few caveats to arming a virus, over an unarmed virus, assuming that the arming device adds to efficacy. Thus, armed replication competent viruses are now the most popular cancer gene therapy approach.

Viruses featuring selective replication in tumor cells, also known as oncolytic viruses, can improve penetration of and dissemination within solid tumor masses [3–5]. Emerging data also suggests their ability to reach distant metastases through vasculature, following release from dying tumor cells [6].

One of the first events during virus replication is amplification of the genome, including the transgene expression cassette, and thus the oncolytic platform allows for high level transgene expression [7, 8]. Further amplification is provided by subsequent cycles of viral replication, release, and infection of more tumor cells. However, a key design aspect is the lysis of the infected tumor cell. If expression on the cell membrane, or inside the cell, is requisite for the transgene product, lysis of the cell might compromise efficacy [8, 9]. Further, expression of molecules with intracellular activity may be superfluous, since the infected tumor cell is expected to die through oncolysis anyway [8]. Thus, oncolytic transgene products should optimally have either paracrine or systemic modes of action.

Historically, oncolytic viruses were intimately associated with infection and immunity. Case reports described tumor regressions following viral infection, with concomitant “flu-like” symptoms. Observations were followed by purposeful contraction of patients, and when toxicity and even mortality were encountered, a less toxic approach was tested, featuring vaccine strains [10]. Following a quiet period of several decades, during which chemotherapy development dominated, oncolytic viruses reemerged in the latter part of the 20th century. This was the era of molecular biology and genetics; the popular laboratory models were cell lines grown in the Petri dish or as xenografts in immunodeficient mice, and thus immunological concepts were overlooked since neither of the popular systems incorporates an intact immune system.

Following high profile descriptions of tumor selective herpes, adenoviral and vaccinia strains [11–13], the field was reinvented by molecular biologists and oncologists. The former attempted to design highly selective viruses to be tested in rigorous *in vitro* experiments, while the latter wanted to just put the viruses into patients and see if tumors would disappear. Modern trial regulations, however, had established a barrier between the bench and the bedside, and thus the key scientists never met any patients treated with their virus, while treating physicians did not usually understand the science in a profound manner. Thus, the flow of information was compromised. The blind were leading the deaf and vice versa.

Scientists like to profile their work as “paradigm shifting,” and when exciting laboratory results did not lead to patient tumors regularly melting away in oncolytic virus trials performed at the turn of the millennium, the “experts” decided that “oncolytic viruses do not work.” Although one or two pioneering clinicians, who understood the science and had treated the patient, attempted to voice their opinions [14], nearly a decade passed before the community started realizing that tumor size may not be a good measure of the activity of oncolytic viruses, especially when armed with immunostimulatory transgenes [15]. No one is Pope in their own land and thus it required Big Pharma data with anti-CTLA4 antibodies [16], a potent form of immunotherapy mediated by downregulation of inhibitory circuits, before “the experts” realized that inflammatory “pseudoprogression” might apply also to oncolytic viruses, resulting in the conclusion that efficacy evaluation should not depend on tumor size measurements alone. However, this required realization that immunology plays a role in therapy with oncolytic viruses [17]. And this data could not be obtained with immunodeficient mice.

In 2005, I was advised by the Finnish Medicines Agency that if my goal was to treat patients, and not to do drug development, I could treat patients, even with drugs not yet approved for sale [18]. Following two years of infrastructure development, production validation, and all kinds of testing, personalized oncolytic virotherapy was started in 2007, in the context of the Advanced Therapy Access Program. It did not take many patients to realize that a lot was going on immunologically. *Rubor*, *color*, *ardor*, and* tumor*, as described by Celsus in 47 BC, were seen. Particularly relevant in the context of assessing efficacy was *tumor*, that is, swelling. If the virus replicated and caused inflammation, the cancer might initially be larger than before treatment, but this might not mean lack of efficacy [15]. One could even postulate that inflammation associated danger signals could associate with efficacy [3].

However, in 2007, few people in the oncolytic virus community had any immunological vision. Even in our laboratory, it required an immune-inspired approach for us to realize that immunology is relevant for all oncolytic viruses [17, 19–25]. While we were the first to describe that oncolytic viruses work in part through induction of an immune response, many others have since agreed [26, 27]. Indeed, there seems little doubt that “dangerous” cell death, such as oncolysis, triggers pathogen associated molecular pattern receptor signaling, resulting in reduction of tumor-induced immunotolerance [3, 28]. With regard to adenovirus, one of the most popular oncolytic platforms, even some mechanisms have been identified [28]. Adenovirus is recognized by pathogen sensing receptors such as TLR9, which leads to “danger signaling,” which is critical for immunity versus tolerance [28].

Unfortunately, it is quite difficult to study the immunology of oncolytic viruses. Many of these viruses are quite species specific, and even if a degree of semipermissivity has been proposed for certain exotic laboratory models such as adenovirus in Syrian hamsters [29], immunological consequences typically differ between different animals. Also, it is fairly obvious that a tumor grown for 10 days in a laboratory animal cannot represent the level of immunosuppressiveness and evasiveness that human tumor has acquired over a decade. Immunological signaling molecules typically feature even more species specificity, often being completely species incompatible, and thus armed viruses are even more challenging to study in the laboratory. Moreover, laboratory reagents are typically scarce when moving beyond human and murine substrates and thus some of the more uncommon models, such as Syrian hamsters, pose analytical problems [29].

Thus, human data has a prominent role in understanding oncolytic viruses. In this regard, it is unfortunate that very few oncolytic virus trials have collected samples for immunological analyses. Not only does the immunotherapeutic potential of most viruses remain poorly understood, but also we lack critical information on mechanisms of action, which complicates optimal administration of the agents and combination with other regimens. An ongoing problem in the field is the disconnect between business management and understanding of the science, leading to poorly informed clinical development decisions and suboptimal trial design, which in turn is counterproductive to the business, slowing down the drug development progress. Ultimately, these issues complicate and delay patients' access to new therapeutics.

There is no dispute that preclinical studies utilizing oncolytic viruses for treating cancer have been highly promising. In contrast, data from clinical trials has been more complicated to interpret [3]. While there is no disagreement that the safety of these approaches has been very good, variability in the frequency of tumor size reductions, typically measured at early time points, has discouraged some analysts [15]. However, possibly the success of other immunotherapeutics will propagate understanding of immunological pseudoprogression and allow “experts” and regulators alike to take mechanistic aspects into account.

Clinical trial results with oncolytic viruses indicate that while single-agent efficacy is seen, striking tumor reductions are relatively few. A likely reason is that early trials typically feature patients with advanced high volume disease refractory to routine therapies. Such tumors are able to rapidly develop resistance to any therapeutic, and unfortunately this extends to oncolytic viruses and other immunotherapeutics, implying that also conventional drugs might have immunological effects. In other words, tumors resistant to chemotherapy and “targeted therapies” are also more immunoedited and immunosuppressive than naïve tumors. One common resistance mechanism may be upregulation of interferon signalling, intriguingly not by the tumor cells themselves, but evidently by the tumor stroma [30]. Moreover, emerging data suggests that pathways responsible for resistance to apoptosis are also involved in immunity.

These considerations constitute a striking example of the low predictive power of laboratory models. There are few or no animal models which would be fully compatible with all the relevant aspects of oncolytic viruses: replication permissiveness, innate and adaptive immunity, activity of the transgene, human tumor tissue, human immunological cells, and so forth. Thus, more than ever, now that the field is maturing towards routine use, it remains critical to obtain human data.

To summarize our own learning curve, which especially in the latter part has been strongly influenced by human data from the Advanced Therapy Access Program [18] but initially began as a laboratory project, we initially thought that safety would be an issue and consequently progressed from prototype, relatively low-selectivity viruses to more selective variants but then proceeded to agents designed for maximum efficacy.

The first generation of oncolytic adenoviruses we studied is embodied by, for example, “delta-24,” a beautifully simple virus with just one modification, a 24-base pair deletion in the E1 gene, which gives the virus selectivity towards the p16/Rb pathway [19, 31]. In the laboratory, at least, it is difficult to infect advanced tumor specimens with unmodified serotype 5 adenovirus, which initiated the field of adenovirus targeting, where capsid modifications, or secretory adapters, are used to enhance gene delivery [32, 33]. The biology behind the phenomenon is that the serotype 5 receptor, the Coxsackie-Adenovirus Receptor, is an adhesion molecule and many adhesion properties are abnormal in advanced tumors [34].

Preclinical considerations and natural caution dictated that safety was foremost, and thus we and many others proceeded to enhance the selectivity of oncolytic adenoviruses [35]. One step in this direction was utilization of tumor specific promoters, which exert their effect prior to E1 (the first gene activated when adenovirus replicates) expression, in contrast to “delta-24” type viruses [36]. Thus, when the promoter is inactive, as in most normal tissues, no E1 expression results, leading to less adenoviral materials in normal cells, in comparison to deletion-mutant viruses, whose selectivity is mediated at steps after E1A expression. “Promoter-bashing” became a field in its own right, aiming at optimizing complex and often large genomic promoter areas into compact fragments that could be used in virus construction [37].

To scientists it was logical that the next step would be to combine promoters and deletion mutants and then to combine this with capsid modification to create “an optimal oncolytic adenovirus” according to the contemporary information [38]. In fact, a triple modified virus appealing in many ways in the laboratory was the first virus we took into patients in the Advanced Therapy Access Program (ATAP) [38–40].

With safety established, but not all patients benefiting from treatment, improving efficacy became top priority. Cautiously, with patient safety foremost in mind, we took a step back and went back to a nonmodified capsid, but this time the virus was armed with granulocyte-macrophage colony stimulating factor, GMCSF. Treatments with this virus were safe and in some patients tumors disappeared [17] while survival also appeared to be promising.

The logical next step to improve patient benefit was to enhance gene delivery with capsid modification, and two approaches were utilized in this regard. Taking the fiber knob from serotype 3 adenovirus and placing it into the serotype 5 capsid allow avoiding the problematic Coxsackie-Adenovirus Receptor, which is often downregulated in advanced tumors [41]. This design was then improved by adding GMCSF, resulting in a potent triple modified virus, Ad5/3-D24-GMCSF [19].

Human data proved to be highly exciting and eventually 115 patients were treated in ATAP [24]. The serotype 3 knob binds to desmoglein 2, which is also an adhesion molecule, seemingly similar to the Coxsackie-Adenovirus Receptor, but in fact desmoglein 2 is not downregulated during carcinogenesis [42]. A variant of this approach utilized incorporating of alpha-v-beta integrin binding RGD-4C in the adenovirus fiber HI-loop [20, 31, 43]. Integrins are adhesion molecules again reminiscent of the usual adenovirus serotype 5 receptor, but as desmoglein 2 they are not downregulated during carcinogenesis [44]. This capsid modification proved to be safe in patients [23], and especially with GMCSF arming, efficacy was also seen [20].

Taking the desmoglein 2 binding approach further, we constructed a fully serotype 3 based oncolytic adenovirus, which proved to be safe in patients, and some efficacy was also seen, even in the absence of arming [21]. Intriguingly, intravenous administration was employed in some patients, resulting in signs of efficacy, especially when combined with monoclonal antibodies. The scientific rationale for the combination is that binding to desmoglein 2 opens tight junctions which enhances the effect of many types of anticancer therapy, including monoclonal antibodies [45]. An attractive next step would be arming of the serotype 3 adenovirus [21, 46].

The 5/3 chimerism approach was taken to yet another level by combining a tumor specific promoter (E2F) and the “delta-24” deletion with the GMCSF expression cassette. This quadruple modified design proved to be highly compatible and resulted in a large proportion of treated patients benefiting [47].

Although GMCSF has many appealing characteristics, a possible caveat is its effects of myeloid derived suppressor cells [17, 19]. This is one reason why we have been interested in also other transgenes, such as CD40 ligand (CD40L), a multifunctional protein which can cause apoptosis of tumor cells, but it can also deactivate suppressive circuits including regulatory T-cells. Moreover, it can modulate the tumor microenvironment from a T-helper type 2 towards a T-helper type 1 situation, with the latter being more conducive to cellular immune responses [48, 49]. Another embodiment of combining the “gas pedal” of oncolysis with “releasing the brake” of immunosuppressiveness is arming the virus with an anti-CTLA4 antibody [50]. As suggested by animal data, this approach might also be appealing from the perspective of systemic toxicity versus local efficacy, as antibody production at the tumor seems to result in favorable distribution. In essence local production restricted to the tumor might enhance antitumor efficacy while reducing systemic toxicities.

Utilizing the capability of sodium iodide symporter hNIS to concentrate radioiodide in tumors is in theory a highly appealing approach which could be in theory used in the treatment of almost any tumor type [51, 52]. The rationale is “plagiarized” from the treatment of thyroid cancer with radioiodide. Thyroid cells naturally express hNIS but the cDNA can be placed into a virus, resulting in transgene expression in any cell allowing virus replication, which is tumor cells in the case of tumor selective oncolytic viruses. However, although the preclinical data was highly promising [51, 52], it was not until we treated the first patient that we realized that very little radioiodide accumulation was seen in tumors. We think this is because the time window between transgene expression and tumor cell lysis is too small to allow concentration of radioiodide to such a degree that could be detected with the most sensitive imaging techniques available [9, 52].

Others have used a virus construct which features less replication, increasing the time window for hNIS expression, and they have been able to detect radioiodide accumulation in the injected tumor. However, following dosimetry calculations, even these authors reported that they were a long way away from therapeutic doses [53]. These depressing results might be caused in part by lack of organification (“incorporation”) of iodide in nonthyroid tissues; even if iodide is transported inside, it will leak out.

Of the 10 viruses used in the Advance Therapy Access Program, Ad5/3-D24-GMCSF, also known as CGTG-102, emerged as the most promising candidate for clinical trials. I cofounded Oncos Therapeutics Ltd. in 2008, and following several years of preclinical development and testing, the company's first clinical trial was started in 2012. Although initially planned as a phase 1-2 trial with an efficacy endpoint, the Finnish regulators (FIMEA) requested restricting the trial to just phase 1. All of the allowed 12 patients have recently completed enrollment and thus trial results are anticipated in 2014. One can assume that the company will then proceed to further trials, possibly aiming at randomized settings, to avoid issues with pseudoprogression and slow response, which are typical of immunotherapy.

Although oncolytic viruses can and do work as single agents, they are appealing for combination with other regimens, as they lack overlap in side effects with, for example, radiation and chemotherapy [54–58]. In the setting of chemotherapy resistant disease, where combination with active dose chemotherapy is not as appealing as with naïve disease, particularly promising combinations include low-dose cyclophosphamide, known to reduce regulatory T-cells, or low-dose pulse temozolomide, an autophagy enhancer. Regulatory T-cells can compromise any immunotherapy approach and since low-dose cyclophosphamide is well tolerated, it was easy to implement this in ATAP [59].

Autophagy induction enhances oncolysis, which in fact is a poorly understood cell death mechanism but may be related to autophagy [54, 60]. There are several publications showing that autophagy inducing agents synergize with oncolytic viruses [25]. Therefore, following preclinical testing and according to the aforementioned scientific rationale, we incorporated low-dose pulse temozolomide into ATAP, for the purpose of enhancing the therapeutic effects of oncolytic viruses, with some promising results [25].

Although many patients have benefited from oncolytic adenovirus treatment, not all did, and in some cases benefits were lost over time despite continued therapy. Thus, it is clear from the clinical data that resistance to therapy can emerge. This phenomenon has not been studied much, but we studied a mouse model which becomes resistant to oncolytic adenovirus and found that interferon response by the tumor stroma (not the tumor cells *per se*!) results in the tumor becoming refractory [30]. Similar findings have been reported for also other oncolytic viruses [61–63]. An immediate conclusion is that anti-interferon approaches might be interesting to enhance the effect of oncolytic virotherapy.

Other means for overcoming the resistance generating capabilities of advanced tumors include treatment of early disease instead of the usual “phase 1 advanced disease population.” In this context, combination with standard therapy is attractive. There are several studies suggesting enhanced cell killing activity when oncolytic viruses have been combined with chemotherapy or radiation [54–58]. In fact, most conventional anticancer approaches can debulk tumor masses, and since large mass correlates with immunosuppressive elements, the efficacy of immunotherapy is consequently enhanced upon debulking.

Moreover, some chemotherapeutics have proposed immunostimulatory activity even on their own [64–67]. Taken together with nonoverlapping side effect profiles, combination regimens are likely to be feasible [68]. However, careful planning, based on human data, is required to optimize regimens in order to reduce immunosuppression without losing the antitumor immunity generated by the virus.

Oncolytic viruses have emerged—or in fact reemerged—as promising thinking-out-of-the-box antitumor agents for treatment of cancer refractory to more conventional treatments. One could argue that immunotherapy is an unutilized sector in oncology, and even if it seems to be entering the mainstream presently with the advent of “passive” monoclonal antibodies against immunosuppressive circuits such as CTLA4 and PD1 [69, 70], active immunotherapeutics such as oncolytic viruses, which can manufacture a personalized cancer vaccine *in situ*, could fulfill an important role currently absent in the antitumor pie chart.

Excitingly, all phase 3 trials completed heretofore seem to support these notions. As often seen nowadays in any sector of society, the Chinese are ahead of the rest, and a decade ago they already completed a randomized phase 3 trial with oncolytic adenovirus H101, in combination with chemotherapy, for treatment of head and neck cancer. Positive results led to approval of Oncorine [71]. In the West, the first randomized trial compared oncolytic herpes virus coding for GMCSF to subcutaneous GMSCF and the primary endpoint was met with a fair margin in 2013. Moreover, progression free survival was improved [72].

Uniquely, oncolytic viruses face complex regulatory and production and intellectual property issues. Overlapping patents, lay concerns over gene delivery, and standardization of biological production systems may be more challenging obstacles than any conceivable scientific issues. Pharmaceutical companies are particularly wary of regulatory requirements for lifelong follow-up of patients, which is not justified by any available data, despite thousands of patients treated. Particularly if treatments prove to be curative, such requirements would result in immense cost.
